# Risk factors for end-stage renal disease in patients with trauma and stage 3 acute kidney injury

**DOI:** 10.1097/MD.0000000000028581

**Published:** 2022-01-21

**Authors:** Kyunghak Choi, Min Soo Kim, Min Ae Keum, Seongho Choi, Kyu-Hyouck Kyoung, Jihoon T. Kim, Sungjeep Kim, Minsu Noh

**Affiliations:** a Department of Surgery, Ulsan University Hospital, University of Ulsan College of Medicine, Ulsan, Republic of Korea; b Department of Neurosurgery, Ulsan University Hospital, University of Ulsan College of Medicine, Ulsan, Republic of Korea.

**Keywords:** end-stage renal disease, renal replacement therapy, traumatic brain injury

## Abstract

Research on long-term renal outcomes in patients with acute kidney injury (AKI) and trauma, especially those with traumatic brain injury (TBI), has been limited.

In this study, we enrolled patients with stage 3 AKI as per the Kidney Disease Improving Global Outcomes guidelines, who initiated renal replacement therapy (RRT). These patients were divided into 2 groups depending on the presence of TBI. Comparing the baseline characteristics and management strategies of each group, we analyzed whether TBI affects the progression of kidney disease.

Between January 1, 2014 and June 30, 2020, 51 patients who initiated RRT due to AKI after trauma were enrolled in this study. TBI was identified in 20 patients, and the clinical conditions were not related to TBI in the remaining 31. The study endpoint was set to determine whether the patients of each group needed RRT persistently at discharge and at the time of recent outpatient clinic. Six (30.0%) out of 20 patients with TBI and 2 (6.5%) out of 31 patients without TBI required conventional hemodialysis, as per the most recent data. No significant within-group differences were found in terms of the baseline characteristics and management strategies. In the logistic regression analysis, TBI was independently associated with disease progression to end-stage renal disease.

TBI is a risk factor for end-stage renal disease in patients with trauma and stage 3 AKI who initiate RRT.

## 1 Introduction

Acute kidney injury (AKI) is one of the most common complications seen in critically ill patients. Several studies have reported that the incidence of AKI in patients with trauma varies from 1% to 50%.^[1–5]^ This broad range is probably due to the heterogeneous AKI criteria used, the differences in the trauma severity, and the length of the follow-up period.^[6]^ Patients with trauma are highly exposed to conditions aggravating kidney injury, such as shock, ischemia, reperfusion, nephrotoxic agents, abdominal compartment syndrome, and direct kidney injury. Emergency laparotomy, interventions, or systemic infections throughout resuscitation trigger these initial AKI risk factors, which leads to impaired renal dysfunction.^[7]^

AKI is associated with prolonged intensive care unit stay and a significantly higher risk of mortality, which increases public health expenses.^[8]^ As such, many studies have been conducted on AKI in patients with severe trauma; however, few studies have focused on long-term outcomes, especially, disease progression to end-stage renal disease (ESRD).

Recent studies proposed that the traumatic brain injury (TBI) is associated with impaired renal function.^[9]^ However, this association between TBI and renal disease progression to chronic kidney disease (CKD) or ESRD is not well-known.

In this study, we classified patients with trauma of stage 3 AKI, as per the Kidney Disease Improving Global Outcomes (KDIGO) guidelines, who initiated renal replacement therapy (RRT) into 2 groups depending on the presence of TBI. Comparing the baseline characteristics and management strategies of each groups, we analyzed whether TBI affects the disease progression.

## 2 Methods

### 2.1 Ethics

This retrospective observational study was based on data obtained from the registry of a single level 1 trauma center. The study protocol was approved by the Institutional Review Board of Ulsan University Hospital (2020-10-027), which waived the need for informed consent.

### 2.2 Study design and population

**Figure 1:**
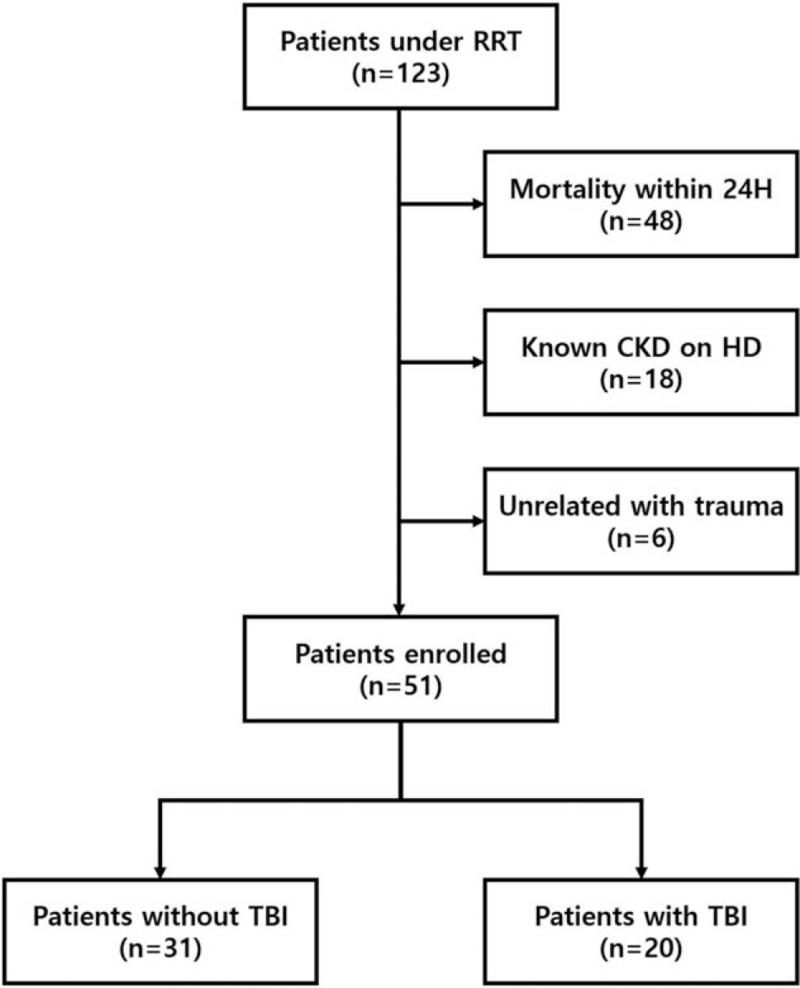
Study enrollment flow diagram. CKD = chronic kidney disease, HD = hemodialysis, RRT = renal replacement therapy, TBI = traumatic brain injury.

Between January 1, 2014 and June 30, 2020, 123 patients underwent RRT due to AKI after trauma, which corresponds to stage 3 AKI according to KDIGO guideline (Table 1). To clarify the long-term outcomes of the enrolled patients, we excluded 48 patients who expired following the discontinuation of life-sustaining treatment within 24 hours. We also excluded 18 patients with a history of known CKD, which might influence the outcome as confounding factors. Moreover, 6 patients whose initial diagnosis was not related to trauma were excluded. Finally, 51 patients were enrolled in this study; TBI was identified in 20 patients, and in the remaining 31, the clinical conditions were not related to TBI. Figure 1 shows the study enrollment flow.

**Table 1:** Staging of acute kidney injury according to the Kidney Disease Improving Global Outcomes guidelines.

Stage	Serum creatinine	Urine output
1	1.5–1.9 times baselineor≥0.3 mg/dL (≥26.5 μmol/L) increase	<0.5 mL/kg/h for 6–12 h
2	2.0–2.9 times baseline	<0.5 mL/kg/h for ≥12 h
3	3.0 times baselineorIncrease in sCr to ≥4.0 mg/dL (≥353.6 μmol/L)orInitiation of renal replacement therapy	<0.3 mL/kg/h for ≥24 horAnuria for ≥12 h

### 2.3 Definitions and study endpoints

RRT includes both continuous RRT and conventional hemodialysis. We administered continuous RRT to all registered patients at the initiation of resuscitative treatment and then converted to hemodialysis after hemodynamic stability was achieved. The primary endpoint of this study was disease progression to ESRD, which was defined as glomerular filtration rate <15 mL/min/1.73 m^2^ or treatment by dialysis for at least 90 days.^[10]^ We examined whether to provide RRT at discharge and at the latest outpatient clinic follow-up point.

### 2.4 Statistical analyses

The categorical variables were represented as frequencies and percentages, and the continuous variables were reported as medians and ranges. The categorical variables were compared using the chi-squared test or Fisher exact test (when the chi-squared test was not available). The continuous variables were compared using the Student *t* test or Mann–Whitney test, based on the distribution of the data. Significant variables (*P* < .25) in the univariate analysis were analyzed by logistic regression for their association with the disease progression. A *P*-value of <.05 was considered statistically significant. All statistical analyses were performed using SPSS version 21.0 (IBM Corp., Armonk, NY).

## 3 Results

### 3.1 Demographic characteristics of the patients

Of the 123 patients, 72 were excluded based on the study criteria, and consequently, 51 were enrolled. The median age was 55 years and 76.5% of the patients were male. The median injury severity score (ISS) was 25 and the median Acute Physiologic Assessment and Chronic Health Evaluation II (APACHE II) score was 26. Of the total study participants, 39.2% had TBI, 33.3% had hypertension, and 7.8% had diabetes. Forty-six patients required emergency surgeries on multiple sites, and 8 out of 51 patients underwent RRT at discharge as the disease progressed. The median number of follow-up was 489 days. The baseline characteristics of the patients are shown in Table 2.

**Table 2:** General characteristics of the patients.

Characteristic		
Age, median (range)	55	(18–84)
Male sex, *n* (%)	39	(76.5%)
ISS, median (range)	25	(4–50)
APACHE II, median (range)	26	(4–41)
TBI, median (range)	20	(39.2%)
HTN, *n* (%)	17	(33.3%)
DM, *n* (%)	4	(7.8%)
HL, *n* (%)	2	(3.9%)
CVA, *n* (%)	3	(5.9%)
CAD, *n* (%)	4	(7.8%)
Malignancy, *n* (%)	3	(5.9%)
Operation, *n* (%)	46	(90.2%)
Head and neck, *n*	16	
Thorax, *n*	8	
Abdominopelvic, *n*	29	
Spine, *n*	2	
Extremities, *n*	24	
RRT at discharge, *n* (%)	8	(15.7%)
Follow-up (d), median (range)	489	(15–2348)

### 3.2 Comparison of the patients depending on the presence of TBI

We divided the patients into 2 groups according to the presence of TBI, and the groups were compared for their demographic and clinical characteristics (Table 3). There was no significant difference in the baseline characteristics between the 2 groups, including age, gender, and premedical history. There was no significant difference in terms of ISS and APACHE II scores. In addition, we compared the worst values of clinically-related laboratory data from admission to during RRT. The laboratory values reflecting kidney function and the inflammatory response showed no differences. Both groups were administered with vasopressors to a similar extent. The serum lactate level was higher in the TBI group; however, the difference was not significant. Two patients in the no TBI group required RRT and 6 patients in the TBI group were undergoing RRT at discharge and the latest outpatient clinic follow-up point, which showed a significant difference (*P* < .05).

**Table 3:** Comparison of patient groups according to traumatic brain injury.

Variables	No TBI (*n* = 31)		TBI (*n* = 20)		*P-*value
Age, median (range)	58	(18–84)	54	(18–77)	.699
Male sex, *n* (%)	24	(77.4%)	15	(75%)	–
ISS, median (range)	22	(5–43)	29	(4–50)	.119
APACHE II, median (range)	27	(5–41)	26	(4–35)	.595
HTN, *n* (%)	10	(32.3%)	7	(35%)	.839
DM, *n* (%)	1	(3.2%)	3	(15%)	.287
HL, *n* (%)	1	(3.2%)	1	(5%)	–
CVA, *n* (%)	1	(3.2%)	2	(10%)	.553
CAD, *n* (%)	2	(6.5%)	2	(10%)	.640
Malignancy, *n* (%)	1	(3.2%)	2	(10%)	.553
Vasopressor, *n* (%)	28	(90.3%)	18	(90%)	–
Lactate (mmol/L), median (range)	4.80	(1.00–14.00)	6.05	(0.90–15.00)	.429
CRP (mg/L), median (range)	26.19	(4.37–46.22)	29.235	(5.19–43.05)	.385
PCT (ng/mL), median (range)	8.38	(0.33–154.99)	6	(0.42–403.90)	.805
BUN (mg/dL), median (range)	85.6	(25.60–183.30)	83.9	(23.90–159.90)	.938
Cr (mg/dL), median (range)	5	(1.38–11.75)	4.62	(1.19–13.11)	.602
GFR (mL/min/1.73 m^2^), median (range)	12	(1.00–57.00)	14.5	(4.00–53.00)	.595
ICU days, median (range)	34	(4–131)	37.5	(3–253)	.582
RRT at discharge, *n* (%)	2	(6.5%)	6	(30%)	.045

### 3.3 Risk factors of persistent RRT

We conducted the chi-squared test or Fisher exact test for categorical variables, and Student *t* test or Mann–Whitney test for continuous variables separately in advance. Significant variables (*P* < .25) in the univariable analysis were analyzed by logistic regression for their association with persistent RRT. Factors known to affect kidney functions and thought to be clinically relevant were also included in the analysis. Table 4 shows that TBI was independently associated with persistent RRT with an increase in the odds ratio of more than 6 times, while the other variables did not reach statistical significance. After confirming that TBI is a risk factor, we conducted a comparative analysis of the entire TBI group depending on the status of persistent RRT, to figure out the factors that made difference in the outcome. Although it was difficult to appreciate statistically significant results due to the small number of patients, the 2 groups showed differences in CRP and GFR, meanwhile there were no differences in the other variables, including the severity of head trauma (Table 5). The use of hydroxyethyl starch and diuretics, commonly known to cause renal dysfunction, was not found in both groups (data not shown).

**Table 4:** Logistic regression analysis of risk factors for persistent renal replacement therapy.

Variables	Odds ratio	95% CI	*P-*value
Age	1.043	0.970–1.121	.253
Male sex	1.597	0.172–14.793	.680
ISS	1.020	0.940–1.106	.634
TBI	6.316	1.054–37.836	.044
HTN	0.749	0.066–8.493	.816
CRP	1.092	0.976–1.221	.123

**Table 5:** Comparison of patients with traumatic brain injury according to the status of persistent renal replacement therapy.

Variables	No RRT (*n* = 14)		RRT (*n* = 6)		*P-*value
Age, median (range)	53.5	(18–76)	64.5	(33–77)	.173
Male sex, *n* (%)	11	(78.6%)	4	(66.7%)	.613
ISS, median (range)	29.5	(4–50)	19.5	(9–43)	.456
APACHE II, median (range)	26	(6–35)	24	(4–29)	.231
AIS, head, median	3.5	(2–5)	4	(3–5)	.391
HTN, *n* (%)	4	(28.6%)	3	(50.0%)	.613
DM, *n* (%)	3	(21.4%)	0	(0.0%)	.521
HL, *n* (%)	1	(7.1%)	0	(0.0%)	–
CVA, *n* (%)	2	(14.3%)	0	(0.0%)	–
CAD, *n* (%)	2	(14.3%)	0	(0.0%)	–
Malignancy, *n* (%)	0	(0.0%)	2	(33.3%)	.079
Operation, *n* (%)	13	(92.9%)	5	(83.3%)	.521
Vasopressor, *n* (%)	12	(85.7%)	6	(100.0%)	–
Lactate (mmol/L), median (range)	6.45	(0.9–15.0)	3.80	(2.06–8.20)	.302
CRP (mg/L), median (range)	27.36	(5.19–39.57)	35.06	(25.78–43.05)	.013
PCT (ng/mL), median (range)	6.00	(0.42–55.80)	7.96	(0.89–403.90)	.888
BUN (mg/dL), median (range)	66.9	(23.9–159.9)	109.3	(60.2–154.9)	.248
Cr (mg/dL), median (range)	3.62	(1.19–8.54)	6.33	(4.46–13.11)	.058
GFR (mL/min/1.73 m^2^), median (range)	17.5	(8–53)	7.5	(4–29)	.002
ICU days, median (range)	37.5	(3–253)	32	(15–69)	.620

## 4 Discussion

Based on previous studies that investigated the relationship between TBI and renal function, we compared and analyzed the enrolled patients according to whether they had TBI. We found that patients with TBI required persistent RRT at discharge and at the latest outpatient clinic points, which was significantly more than in those without TBI. In this study, 8 patients with trauma required RRT, and the sole risk factor of persistent RRT due to disease progression was TBI. All other variables appeared to have no significant difference among patients with or without TBI. Moreover, the 2 groups showed no difference in the APACHE II and ISS scores, implying that the injuries were of the same magnitude.^[11]^ In order to find the cause of the significant difference in the results, we further investigated the treatment strategies of the patient groups, but none of the variables showed a significant difference, including fluid and nephrotoxic drug usage, which was observed only in a single patient of the persistent RRT group.

Although the mechanism of AKI in patients with TBI have not been fully elucidated, it can be considered from 2 main perspectives, trauma and brain injury. First, patients with trauma are highly exposed to conditions aggravating kidney injury, such as hypovolemic shock, frequent use of nephrotoxic agents for diagnosis, and direct kidney injury. Routinely administered medications such as NSAIDS also deteriorate kidney function. Second, brain injury leads to excessive secretion of antidiuretic hormone, causing hyponatremia and fluid imbalance. In addition, stimulated sympathetic nervous activity with increased level of plasma catecholamine, results in hypertension with decreased kidney perfusion.^[10]^ Inflammatory cascades after brain injury converged with complement activation and inflammatory cytokine release. These proinflammatory mediators pass the damaged blood brain barrier into systemic circulation, causing organ injury.^[12]^ Civilett et al^[13]^ carried out an in vitro study with human tubular epithelial cells to clarify the association between severe TBI and acute tubular injury. This study showed that the inflammatory process associated with TBI correlates with renal function. TBI triggers a complex cascade of cellular events, leading to systemic inflammation and damage of kidney and other organs. Civilett et al also mentioned that this hypothesis might be supported by the increased neutrophil gelatinase-associated lipocalin level, which was significantly elevated, even if biomarkers currently used for the diagnosis of AKI appeared to be within the normal range. Although it cannot be considered statistically meaningful due to the small number of subjects, the correlation between inflammatory markers and kidney disease progression also can be glimpsed in the Table 5. Concerning inflammatory markers, it is difficult to explain the significant difference of CRP level using only the data collected in this study. If additional profound research is conducted on the pathophysiology of TBI, the 2 clinical findings that show significant differences, high degree of inflammation and consequent severely elevated eGFR based on the MDRD study, would be the most conceivable candidates of fundamental risk factors. Further studies from this perspective verify the anticipation.

AKI to CKD transition is mainly caused by maladaptive repair of kidney injury. This process includes many pathophysiological processes, for example, cell death or acute tubular necrosis, renal fibrosis, which are characterized by the accumulation of extra cellular matrix, capillary rarefaction by endothelial to mesenchymal transition, tubular epithelial cell senescence, and consequent inflammatory processes.^[14]^ However, the circumstances under which the disease progresses into ESRD has not been precisely evaluated. Nonetheless, a recent study provided clues to estimate the conditions of disease progression.

Wu et al^[10]^ performed a retrospective observational analysis of long-term renal outcomes of patients with TBI and found that patients with TBI developed significantly more CKD than those without; however, there was no difference in the progression to ESRD. The study covered nationwide population of patients with TBI regardless of kidney function, whereas we focused on patients with stage 3 AKI who initiated RRT at the critical phase of management. The estimated reason for the difference in the result comes from the kidney function of enrolled patients at the starting point of analysis. In future studies, significant conclusions about long-term kidney function might be drawn through the analysis in accordance with the presence or absence of TBI in each AKI stage as per KDIGO guideline. These results would be highly versatile in terms of their clinical implications and may enable to anticipate the graft function of the brain-dead kidney donors.^[15]^

A considerable limitation of this study is the heterogeneity in the management; patients with TBI were managed mainly by neurosurgeons and those without TBI were managed by general surgeons. Therefore, consistent treatments were not applied to the study patients. In addition, this study was a retrospective analysis conducted using registry data from a single trauma center and consisted of a relatively small number of patients in each group. Thus, selection and information biases might have affected the results. Moreover, the diagnosis of AKI and initiation of RRT were based on patients’ clinical status judged by diverse physicians. It was also a limitation of this study that long-term follow-up of the patients was not performed.

## 5 Conclusion

TBI is a risk factor for ESRD in patients with trauma who develop stage 3 AKI, as per the KDIGO guideline. TBI patients might benefit by prudent monitoring of kidney function and early detection of disease. Through early initiation of multidisciplinary management, especially with nephrology, it is possible to reduce the possibility of disease progression by restricting clinical conditions that can cause nephrotoxicity while maintaining volume status.

## Author contributions

**Conceptualization:** Kyunghak Choi, Kyu-Hyouck Kyoung, Jihoon T Kim.

**Data curation:** Min Soo Kim, Min Ae Keum, Seongho Choi, Sungjeep Kim.

**Formal analysis:** Kyunghak Choi.

**Methodology:** Kyu-Hyouck Kyoung.

**Project administration:** Minsu Noh.

**Resources:** Kyunghak Choi, Min Soo Kim, Min Ae Keum, Seongho Choi, Kyu-Hyouck Kyoung, Jihoon T Kim, Sungjeep Kim, Minsu Noh.

**Writing – original draft:** Kyunghak Choi.

**Writing – review & editing:** Jihoon T Kim, Minsu Noh.
